# Relationship between osteosarcopenic obesity and dietary inflammatory index in postmenopausal Korean women: 2009 to 2011 Korea National Health and Nutrition Examination Surveys

**DOI:** 10.3164/jcbn.18-10

**Published:** 2018-05-12

**Authors:** Susan Park, Woori Na, Cheongmin Sohn

**Affiliations:** 1 Department of Food and Nutrition, Wonkwang University, Iksan-daero, 460, Iksan, Jeonbuk 54538, Korea

**Keywords:** osteosarcopenic obesity, dietary inflammatory index, inflammation, postmenopausal women, nutrition

## Abstract

Osteosarcopenic obesity syndrome is a condition including osteopenia, sarcopenia and obesity. A pro-inflammatory dietary pattern has been reported to be associated with obesity and osteoporosis. However, studies on the association of dietary inflammatory index with osteosarcopenic obesity syndrome in the Korean population are lacking. The aim of this study was to analyze the relationship between dietary inflammatory index and osteosarcopenic obesity syndrome among Korean postmenopausal women. We analyzed the 2009–2011 Korea National Health and Nutrition Examination Survey, consisting of 1,344 postmenopausal women aged 50 years or older. Body composition was evaluated by dual-energy X-ray absorptiometry. Dietary inflammatory index was estimated after analyzing 36 nutrients and 9 foods using a 24-h dietary recall data. The association between dietary inflammatory index levels and the body composition was analyzed by logistic regression models with dietary inflammatory index fit as a dichotomous variable. The dietary inflammatory index was −0.96 ± 0.22 in the normal group, 0.12 ± 0.16 in the osteopenic obesity group, 0.00 ± 0.18 in the osteosarcopenia group, 0.12 ± 0.33 in the sarcopenic obesity group, and −0.02 ± 0.14 in the osteosarcopenic obesity group (*p*<0.001). After adjusting for potential covariates, women with higher dietary inflammatory index scores were more likely to have risk of osteopenic obesity (OR = 2.757, 95% CI: 1.398–5.438, *p*<0.01) and that of osteosarcopenic obesity (OR = 2.186, 95% CI: 1.182–4.044, *p*<0.05). The results indicate that pro-inflammatory diet was associate with increased odds of the osteosarcopenic obesity in postmenopausal Korean women. Therefore, studies are needed to identify the effects of anti-inflammatory diets, which can reduce the degree of inflammation through dietary intake.

## Introduction

Bone, muscle and fat constituting the body are correlated with each other, and changes in body composition have been found to have an effect on changes in the whole body and surrounding tissues.^(1)^ As humans get older, their body composition changes due to aging, which mainly lead to bone loss, decrease in muscle mass, and increase in body fat.^(2)^ Recently, Korea has seen an increase in the prevalence of obesity due to various factors, such as westernized eating habits resulting from the improvement of living standards, and the decrease in physical activity levels. According to the National Health and Nutrition Examination Survey, obesity prevalence in women, based on body mass index (BMI), was found to be 36.2% among those aged 50–59 years, 41.7% among those aged 60–69 years, and 40.8% in aged 70 or older in 2015. It was found that with increasing age, the prevalence of obesity increases.^(3)^ Particularly in postmenopausal women, as age increases, bone density decreases, which in turn causes osteoporosis.^(2)^

Adipose tissues, previously thought to be simple energy storage warehouses are currently known to function as endocrine organs causing metabolic changes in the body, including inflammatory reactions. In addition, adipose tissues that are increased in obese individuals were previously thought to protect against osteoporosis; however, recent studies have disproved this theory.^(4)^ It was found that increased intestinal and abdominal adipose tissues due to obesity cause the secretion of pro-inflammatory cytokines, such as adiponectin, IL-6, TNF-α, etc., leading to an increased risk of falls and fractures. In particular, it has been shown that increase in inter-muscular adipose tissue that accumulates in the femoral tissue causes reduction in muscle strength, bone mineral density reduction and metabolic diseases.^(5)^ This suggests that excess adipose tissue may cause a loss of bone mineral density and muscle mass and may act as a risk factor for sarco-osteopenia or sarco-osteoporosis symptoms.^(6)^

The serum inflammation index is known to be related to dietary and nutrient intake. In a study of overweight women, levels of adiponectin in the blood that plays an anti-inflammatory role decreased as calories and carbohydrate intake increased, whereas levels of serum adiponectin increased as the dietary fiber and vitamin C increased.^(7)^ In a study of adults with metabolic syndrome, adiponectin decreased as the intake of more lipids, total sugars and total fatty acids increased, and as the intake of polyunsaturated fatty acids increased, high-sensitivity C-reactive protein levels increased.^(8)^ In a study of overweight and obese adults, as protein intake increased, the levels of IL-6, an inflammatory marker in the blood, deceased.^(9)^ In this way, the serum inflammation index has been reported to be associated with dietary and nutrient intake in patients with chronic disease.

The dietary inflammatory index (DII), which can be used to measure the degree of inflammation non-invasively through dietary intake, was developed by Shivappa *et al.*^(10)^ The DII is a tool used to measure the degree of inflammation, and was formulated by analyzing 36 nutrients and 9 foods derived from a systematic review of the literature, including animal experiments and epidemiological studies.^(10)^ Although there are reports that the DII is associated with various diseases such as metabolic syndrome, obesity, and cancer, there has been insufficient research to utilize the DII in Korea. Additionally, there have been few studies on the nutrient intake status and the inflammatory index related to body composition in Korea.

We conducted a cross-sectional study to identify the risk of sarcopenic obesity (SO), osteosarcopenia (OS), osteopenic obesity (OO), and osteosarcopenic obesity (OSO) according to the DII in a representative sample of postmenopausal women by using data from the 2009–2011 National Health and Nutrition Examination Survey.

## Materials and Methods

### Study population

This study was performed using the original data of the National Health and Nutrition Examination Surveys for 2009–2011 (4th–5th period) conducted by the Ministry of Health and Welfare. The National Health and Nutrition Examination Surveys are conducted to assess the health and nutritional levels of the population and to produce the statistics necessary to establish and evaluate national health policies. It was designed to be able to extract a representative sample of Korean individuals with the population and housing census data as a sampling frame, composed of a nutrition survey, a health survey including a health interview and a health awareness survey, and a check-up survey. The nutrition survey was conducted by trained dietitians who visited individual households and interviewed individuals by asking them about their eating habits, frequency of food intake, type of food intake, and quantity of intake. The health survey was performed by survey personnel who visited individual households and interviewed them through a status table of survey targets, a household questionnaire, and a health interview. The check-up survey was conducted by setting up a mobile check-up center in the survey area and taking anthropometric measurements and blood samples. A total of 28,009 individuals (10,533 in 2009, 8,958 in 2010, and 8,518 in 2011) participated in the 4th and 5th National Health and Nutrition Examination Surveys. We excluded all 12,825 men, and cases with missing values in the survey, anthropometric measurements, biochemical analysis, and bone density measurement data. Additionally, those diagnosed with stroke, myocardial infarction, angina pectoris, liver cirrhosis, renal failure, and cancer were excluded. In addition, those with calorie intakes of 500 kcal and less and 4,000 kcal and more were also excluded. Thus, a total of 1,344 postmenopausal women older than 50 years were included in the final analysis. The present study was approved by the Clinical Test Deliberation Commission of Institutional Review Board (IRB), Wonkwang University (WKIRB-201801-SB-007).

### General characteristics

The anthropometric characteristics of the subjects included heights, weights, waist circumferences, body mass index, appendicular skeletal muscle mass, and body fat percentages collected through a check-up survey. For the bone mineral density, appendicular skeletal muscle mass and total body fat percentage, the data measured by dual-energy X-ray absorptiometry (DXA), a bone mineral density meter (Discovery W, Hologic, Bedford) were used. For systolic and diastolic blood pressure, the mean value was used after three measurements in a stable state. Biochemical indicators included total cholesterol and blood vitamin D levels. For the nutrient intake status, analysis was made, based on the data provided in an individual 24 recall.

### Diagnostic criteria of sarcopenia and obesity

 Sarcopenia was diagnosed if the percentage-applied value acquired by dividing appendicular skeletal muscle mass (ASM) by weight, expect for the bone and fat measured by DXA, was less than 1 SD below the average of women aged 20–40 years participating in the National Health and Nutrition Examination Surveys. As a result of that, the cut-off value of sarcopenia in this study was defined as 21.21%. The determination of obesity was made by classifying body mass indexes (BMI) into normal weight (18.5 kg/m^2^≤BMI<23 kg/m^2^), overweight (23 kg/m^2^≤BMI<25 kg/m^2^), and obesity (BMI>25 kg/m^2^).

### Osteoporosis diagnosis criteria

For bone mineral density, data were used, which were acquired from the data of the bone densities measured on the entire femur, femoral neck, and lumbar spine using DXA. Osteoporosis diagnosis criteria were based on the T-scores (values indicating standard deviation), compared to the average of young people of the same sex and ethnicity defined by the World Health Organization. The diagnosis was made by classifying the T-scores into the normal group, the osteopenia group (−1>T-score>−2.5), and the osteoporosis group (−2.5≥T-score).^(11)^

### Analysis of the DII

For the analysis of the DII, standardized values were calculated using the mean and standard deviation of the data from 11 countries (USA, Australia, Bahrain, Denmark, India, Japan, New Zealand, Thailand, Korea, Mexico and Britain) presented in the study of Shivappa on 6 foods and 36 nutrients through a 24-h dietary recall.^(10)^ To minimize the biases of foods and individual nutrients for the DII, the DII score was calculated by multiplying the percentile score and sum of each food and nutrient. The items of the DII applied in this study include the nutrients of foods, such as garlic, ginger, onion, turmeric, green tea/black tea, pepper, and others. The items also cover the nutrients of alcohol, caffeine, carbohydrate, protein, cholesterol, energy, fat, dietary fiber, vitamin B_12_, vitamin B_6_, β-carotene, folic acid, iron, magnesium, monounsaturated fatty acids, polyunsaturated fatty acids, niacin, omega-3 fatty acids, omega-6 fatty acids, thiamine, riboflavin, saturated fatty acids, trans fats, selenium, vitamin A, vitamin C, vitamin D, vitamin E, zinc, anthocyanidins, flavan-3-ols, flavanones, flavones, flavonols, isoflavones, and others. A low DII represents a meal rich in anti-inflammatory components and *vice versa*.

### Statistical analysis

For the data of individuals registered in the National Health and Nutrition Examination Survey, health or check-up and nutrition weights were applied to represent the entire population of Korea. These data were then analyzed by multiplying the surveyed population ratios by year and calculating the integrated weights after integrating bone density and body fat examination data for 2009–2011. The continuous variables of the subjects used in this study were expressed as mean ± SE by performing analysis of variance (ANOVA) of the general linear model. The nominal variables were expressed as frequency and percentage (%) by performing a crossover analysis. The subjects were divided into 5 groups: the normal, osteopenic obesity, osteosarcopenia, sarcopenic obesity, and Osteosarcopenic obesity groups. Correlation with the continuous variables on general characteristics was analyzed using Pearson’s correlation coefficient. For the analysis of differences between the 5 groups according to general characteristics, a composite-sampled linear model was used. For the risk analysis of each symptom group according to DII, multivariate logistic regression analysis was conducted and the variables of age, income level, regular exercise, education level, smoking status, and whether or not to use female hormone were calibrated to calculate an odds ratio (OR) and a 95% confidence interval (CI). Statistical processing of all data was done using SPSS ver. 21.0 (IBM Corp., Armonk, NY). Statistical significance was set *p*<0.05.

## Results

Table 1 summarizes the general characteristics of the study subjects. The subjects of this study comprised 11.6% (*n* = 131) in the normal group, 24.2% (*n* = 334) in the OO, 24.1% (*n* = 314) in the OS, 8.2% (*n* = 110) in the SO, and 31.8% (*n* = 455) in the OSO. The mean age of the subjects was significantly higher (63.99 ± 0.50) in the OSO than the other groups (*p*<0.001). The mean total cholesterol levels of the subjects was significantly higher 211.58 ± 3.51 mg/dl in the SO than in the other groups (*p*<0.01). As a result of the nutrient intake analysis, average calorie intake of subjects did not show difference in all groups. However, the SO, OS, OO, OSO scored significantly lower than the normal group regarding the intake of vitamin C (*p*<0.01) and vitamin E (*p*<0.05).

Table 2 shows the results of the comparative analysis of the difference in the DIIs according to the adverse body composition. The DII was −0.96 ± 0.22 in the normal group, 0.12 ± 0.16 in the OO, 0.00 ± 0.18 in the OS, 0.12 ± 0.33 in the SO, and −0.02 ± 0.14 in the OSO. Compared to other groups, the SO scored significantly higher in the DII (*p*<0.001).

The association of DII with adverse body composition was examined (Table 3). Subjects who scored over the median DII showed a higher prevalence of OO (OR = 2.757; 95% CI: 1.398–5.438) (*p*<0.01) and OSO (OR = 2.186; 95% CI: 1.182–4.044) (*p*<0.05) after adjusting for potential confounders.

## Discussion

This study tried to compare the DII in the normal, OSO, OO, OS and SO groups using the data of the 2009–2011 National Health and Nutrition Examination Surveys. The present study also attempted to analyze the risk of each symptom according to the DII.

In this study, a method of dividing limb muscle mass by body weight was used. Through these criteria, OSO was diagnosed, where obesity, osteopenia, and sarcopenia appear at the same time. In this study, the prevalence of OSO in postmenopausal women over 50 years of age was 31.8%. In a study of limb muscle mass divided by height in women over 50 years of age according to the 2008–2010 National Health and Nutrition Survey, the prevalence of OSO was 25%.^(12)^ In a study in which body function, strength, and skeletal muscle mass were considered, based on the criteria of the European Working Group on Sarcopenia in Older People, the prevalence of OSO was 12%.^(13)^ As the result of a study of Mexican women older than 50 years considering appendicular lean mass, based on the criteria of the Foundation for the National Institutes of Health, the prevalence of OSO turned out 19%, showing some difference from that of this study.^(14)^ Changes in the body composition of elderly people due to aging, loss in body fat, bone mass, and muscle mass may appear. There have been various studies on the criteria of each index for diagnosing the changes of body composition. For the diagnosis of obesity, a body fat percentage test through DXA or BIA and a ratio of hip-waist circumference through body measurement, or BMI have been set as a typical diagnostic criterion. For the diagnosis of osteopenia or osteoporosis, the t-score through DXA diagnosis is a representative indicator. However, the criteria for sarcopenia is highly influenced by race and physique, so there is a difference between the diagnostic method and the cut-off value according to the study. When sarcopenia is diagnosed by dividing the limb muscle mass by body weight, there may be an error in selecting the subjects with low muscle mass and high body weight. For the method of dividing the limb muscle mass by height, it has been reported that the prevalence of sarcopenia may increase, so it is presumed that there is a difference in prevalence among the OSO groups including sarcopenia for each study.^(15,16)^ There has been no definite diagnostic criteria for sarcopenia. Thus, it is thought that a consistent diagnosis of OSO should be made by establishing the diagnostic criteria for sarcopenia, applicable to Korean adults.

Recently, studies have shown that increased fat in obesity reduce bone density and increases the risk of osteoporosis. Thus, contrastive studies on a theory of osteoporosis defense of fat have been reported. The analysis of postmenopausal women in Korea showed BMI was negatively correlated with BMD. ^(17)^ In addition, as a result of analyzing Chinese postmenopausal women, fat content and bone mineral content were negatively correlated. It was found that as the body fat percentage increases, the risk of osteoporosis increases.^(18)^ Through the reported mechanism of this phenomenon, mesenchymal stem cells are differentiated into osteoblasts, osteocytes, adipocytes and muscle cells immune-related cells as well as immune-related cells. The increase in excess fat tissue in obese men leads to the secretion of pro-inflammatory cytokines such as leptin, adiponectin, IL-6, TNF-α, CRP, and others. This inhibits osteoblast activity and muscle cell formation, resulting in bone and muscle loss.^(19)^ Thus, it is found that bones, muscles, and adipose tissues considered to be target organs that simply act on hormones secrete a variety of hormones and have an effect on the whole body. There is a need for in-depth studies on the correlation among body organizations and osteopenia/osteoporosis, sarcopenia, and obesity.

Pro-inflammatory cytokines expressed in fat cells have been shown to affect the development of chronic diseases.^(5)^ Thus, the inflammatory index is reported to be useful as an indicator for predicting the risk of developing a chronic diseases.^(20)^ The DII is a tool developed by Shivappa *et al.*^(10)^ which can be used to measure the degree of inflammation through the correlation analysis between pro-inflammatory cytokines and meal intake. In this study, the DII of the OSO, OO, and OS groups were found to be higher than that of the normal group. As a result of analyzing nutrient intake, the intake of vitamin C and vitamin E as antioxidant nutrients was significantly lower in all the groups than the normal group. In a study of urban-based cohort data for women older than 40 years, as the number of diagnoses of metabolic syndrome increased, the DII was higher.^(21)^ In addition, in a study of women aged 60–80 years from the Prevencion Con Dieta Mediterranea (PREDIMED) of Spain, BMI and WHR related to obesity showed a significant difference from the DII. Thus, the result was found to be similar to that obtained in this study.^(22)^

The analysis of the risk of each symptom according to the DII showed that as the DII increases, the risks in the OSO, OO, OS, and SO groups increases. In a study analyzing the association between the DII and metabolic syndrome in Koreans enrolled in city-based cohort data, as the DII increased, the risk of metabolic syndrome increased. The study also found that as the number of metabolic syndrome judging indices increases, the risk of metabolic syndrome increases.^(21)^ In a study of the relationship between the DII and colorectal cancer in Koreans, it was found that the risk of colorectal cancer increased as the DII increased. This study also reported that if the smoking status continues with age increasing and physical activities lacking, the DII is high.^(23)^ In addition, as the DII increased, the risk of obesity was high in women aged 60–80 years in the PREDIMED of Spain. Thus, it was found that as the DII increases, the risk of cardiovascular disease increases.^(22)^ Westernized eating habits and lifestyle changes due to the improvement of living standards contribute to the increase in the prevalence of obesity and nutritional imbalance. Especially, when vitamin C, known as an antioxidant and an anti-inflammatory nutrient is deficient, bone remodeling through collagen biosynthesis becomes inactivated.^(24)^ This in turn reduces bone mass and increases the risk of fracture. In addition, it has been reported that when Vitamin E with an antioxidant function is deficient, osteoclasts are activated due to oxidative stress and inflammation, which then reduces bone mass and causes fat to penetrate into muscle cells.^(24,25)^ In other words, unbalanced nutrient intake can lead to high levels of DII and act as a risk factor for osteopenia/osteoporosis, sarcopenia, and obesity. Therefore, for healthy aging and falling risk and injury prevention, it is important to keep balanced nutrient intake and anti-inflammatory nutrient intake and meals. Further studies are needed on the interaction of fat, muscle, and bone, which affect OSO for the prevention and treatment of OSO.

The limitations of this study include the following: First, this study was conducted in a form of a cross-sectional study using the National Health and Nutrition Examination Surveys so it was impossible to analyze the causal relationship between factors affecting bone, muscle, adipose tissue and inflammatory markers associated with cardiovascular diseases. In addition, conditions of walking abilities, grip strength, etc. were excluded so it was impossible to make a clear diagnosis. Second, the 24-h recall method used in this study is dependent on one’s memory of the type of eaten food and the intake 1 day before the survey. Additionally, it may be difficult for the elderly to remember the exact type of food and intake and individual diets change daily. Thus, the method might have not reflected daily nutrient intake. Third, this study was limited to females. Therefore, there is a limit in that it is impossible to figure out whether or not to apply the risk factors of OSO resulting from this study to males as well. Despite these limitations, this study analyzed the relationship between OSO and DII using the National Health and Nutrition Examination Surveys data and the results seem to be meaningful as the data may be representative of postmenopausal Korean women older than 50 years. Considering the results of this study, the intake of desirable nutrient and the maintenance of muscle mass and healthy weight are needed to prevent osteoporosis and sarcopenia which can result from the increase in excessive body fat. In future, it is important to determine the effectiveness of anti-inflammatory diets to reduce inflammation through clinical and prospective studies rather than cross-sectional studies. It is expected that the results of this study can be utilized as the basic data on meal intake and DII in OSO in the future.

## Figures and Tables

**Fig. 1:**
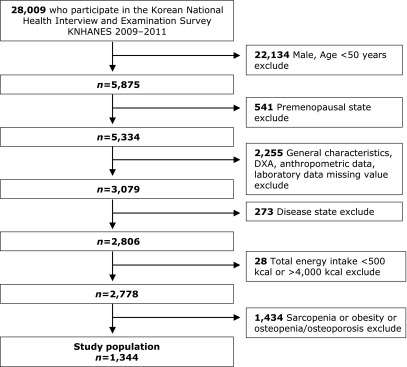
Flowchart of participant inclusion in the study.

**Table 1:** The general characteristics of the study subjects^†^

Characteristic	Total	Normal	Sarcopenic Obesity	Osteosarcopenia	Osteopenic Obesity	Osteosarcopenic Obesity	*p* value
*n* (%)	1,344	131 (11.6)	110 (8.2)	314 (24.1)	334 (24.2)	455 (31.8)	
Age (years)	62.34 ± 0.31	56.72 ± 0.47	61.42 ± 0.85	62.75 ± 0.69	63.21 ± 0.59	63.99 ± 0.50	<0.001
Body mass index (kg/m^2^)	25.70 ± 0.09	22.85 ± 0.13	28.12 ± 0.35	23.77 ± 0.14	26.24 ± 0.13	27.15 ± 0.17	<0.001
Smoker							<0.001
nonsmoker	1,254 (92.4)	125 (95.6)	107 (96.6)	289 (88.4)	308 (92.2)	425 (93.3)	
past smoker	18 (1.4)	1 (0.8)	0 (0.0)	5 (2.7)	4 (1.0)	8 (1.4)	
current smoker	72 (6.2)	5 (3.5)	3 (3.4)	20 (8.9)	22 (6.7)	22 (5.3)	
Education							<0.001
≤Elementary	858 (61.2)	36 (26.1)	69 (64.0)	188 (59.5)	247 (71.7)	318 (66.6)	
Middle school	210 (17.7)	27 (24.0)	19 (16.6)	45 (16.5)	52 (17.3)	67 (16.8)	
High school	214 (16.6)	48 (35.8)	15 (13.2)	64 (19.9)	30 (9.8)	57 (13.3)	
≥University	59 (4.4)	20 (14.0)	5 (6.2)	16 (4.2)	5 (1.1)	13 (3.3)	
Family income							<0.001
Low	456 (30.8)	22 (16.6)	44 (38.0)	92 (25.9)	134 (36.3)	164 (33.6)	
Moderate-low	359 (27.3)	29 (21.4)	17 (11.6)	95 (34.0)	98 (28.8)	120 (27.4)	
Moderate-high	260 (21.3)	28 (24.1)	26 (24.4)	59 (19.9)	58 (18.6)	89 (22.3)	
High	259 (20.6)	50 (37.9)	23 (26.1)	64 (20.1)	44 (16.3)	78 (16.7)	
Exercise: moderate							0.032
Yes	185 (13.5)	17 (16.0)	9 (7.0)	42 (12.9)	56 (16.1)	61 (12.7)	
No	1,159 (86.5)	114 (84.0)	101 (93.0)	272 (87.1)	278 (83.9)	394 (87.3)	
Body composition							
ASM (kg)	13.98 ± 0.07	14.07 ± 0.19	14.77 ± 0.19	12.63 ± 0.98	14.89 ± 0.19	14.06 ± 0.10	<0.001
ASM/Wt (%)	23.16 ± 0.08	24.92 ± 0.26	22.18 ± 0.14	22.55 ± 0.06	24.21 ± 0.19	22.43 ± 0.12	<0.001
Total body fat mass (%)	37.58 ± 0.17	33.23 ± 0.46	40.03 ± 0.44	37.85 ± 0.22	36.53 ± 0.38	39.14 ± 0.27	<0.001
BMD (g/cm^2^)							
Whole body total	1.03 ± 0.01	1.14 ± 0.09	1.07 ± 0.02	1.01 ± 0.01	1.00 ± 0.01	1.02 ± 0.01	<0.001
Total femur	0.80 ± 0.00	0.91 ± 0.01	0.85 ± 0.02	0.77 ± 0.01	0.78 ± 0.01	0.78 ± 0.01	<0.001
Femoral neck	0.64 ± 0.00	0.76 ± 0.01	0.70 ± 0.01	0.62 ± 0.01	0.62 ± 0.01	0.63 ± 0.01	<0.001
Lumbar spine	0.84 ± 0.00	0.98 ± 0.01	0.88 ± 0.02	0.81 ± 0.01	0.78 ± 0.00	0.83 ± 0.01	<0.001
Laboratory data							
SBP (mmHg)	129.45 ± 0.60	123.67 ± 1.87	133.88 ± 1.55	129.32 ± 1.31	129.23 ± 1.02	130.69 ± 1.02	<0.001
DBP (mmHg)	79.53 ± 0.35	78.82 ± 1.18	81.99 ± 0.95	78.85 ± 0.74	79.46 ± 0.60	79.67 ± 0.61	<0.001
Total-cholesterol (mg/dl)	205.91 ± 1.16	199.49 ± 3.51	211.58 ± 3.51	206.05 ± 2.38	203.8 ± 2.39	208.49 ± 2.23	0.002
Vitamin D (ng/ml)	17.55 ± 0.26	17.04 ± 0.59	17.47 ± 0.64	17.62 ± 0.47	18.36 ± 0.52	17.11 ± 0.39	0.153
Nutrient intake							
Energy (kcal)	1,551.52 ± 19.68	1,634.10 ± 48.72	1,618.51 ± 82.36	1,534.91 ± 42.94	1,543.40 ± 31.79	1,522.77 ± 37.15	0.380
Protein (g)	53.35 ± 0.92	59.80 ± 2.66	54.12 ± 3.83	53.53 ± 1.94	50.77 ± 1.37	52.63 ± 1.81	0.061
Calcium (mg)	441.34 ± 15.69	482.99 ± 27.00	429.73 ± 35.82	447.33 ± 21.12	389.66 ± 15.82	465.85 ± 43.94	0.167
Vitamin C (mg)	96.75 ± 2.81	129.68 ± 10.26	97.71 ± 9.90	99.34 ± 7.23	87.12 ± 4.32	89.84 ± 3.92	0.004
Vitamin E (mg)	10.54 ± 0.53	11.84 ± 0.85	10.05 ± 1.07	12.16 ± 2.00	9.21 ± 0.38	10.03 ± 0.61	0.040
Female hormone supplements							0.001
Yes	247 (17.5)	39 (32.0)	18 (14.3)	63 (17.8)	52 (15.2)	75 (14.5)	
No	1,097 (82.5)	92 (68.0)	92 (85.7)	251 (82.2)	282 (84.8)	380 (85.5)	
Calcium supplements							0.077
Yes	246 (17.7)	32 (23.4)	14 (10.5)	71 (21.2)	52 (14.3)	77 (17.5)	
No	1,098 (82.3)	99 (76.6)	96 (89.5)	243 (78.8)	282 (85.7)	378 (82.5)	
Age of menopause (years)	49.31 ± 0.16	49.94 ± 0.54	49.22 ± 0.65	49.01 ± 0.37	49.55 ± 0.31	49.19 ± 0.24	0.205

ASM, appendicular skeletal muscle mass; BMD, bone mineral density; SBP, systolic blood pressure; DBP, diastolic blood pressure. ^†^Values are presented as mean ± SE or percentage.

**Table 2:** DII according to adverse body composition^†^

	Normal (*n* = 131)	Osteopenic obesity (*n* = 334)	Osteosarcopenic (*n* = 314)	Sarcopenic obesity (*n* = 110)	Osteosarcopenic obesity (*n* = 455)	*p* value
DII Score^‡^	−0.96 ± 0.22	0.12 ± 0.16	0.00 ± 0.18	0.12 ± 0.33	−0.02 ± 0.14	0.001

^†^Values are mean ± SE. ^‡^DII score range: −6.37~6.55.

**Table 3:** Analysis of risks by symptom according to the DII

Variables	DII (With <median as referent)	Crude OR (95% CI)	*p* value	Adjusted OR (95% CI)^†^	*p* for trend
Normal vs osteopenic obesity	≤−0.07	1.00 (reference)	<0.001	1.00 (reference)	0.004
	>−0.07	2.952 (1.756–4.963)		2.757 (1.398–5.438)	

Normal vs osteosarcopenic	≤−0.07	1	<0.001	1	0.153
	>−0.07	2.706 (1.628–4.497)		1.565 (0.845–2.898)	

Normal vs sarcopenic obesity	≤−0.07	1	0.031	1	0.068
	>−0.07	2.169 (1.085–4.334)		1.968 (0.951–4.073)	

Normal vs osteosarcopenic obesity	≤−0.07	1	<0.001	1	0.013
	>−0.07	2.067 (1.121–3.810)		2.186 (1.182–4.044)	

OR (95% CI); Odds ratios (95% confidence interval). DII score range: −6.37~6.55. ^†^Adjusted for age, family income, regular exercise, education status, smoking and female hormone supplements.
